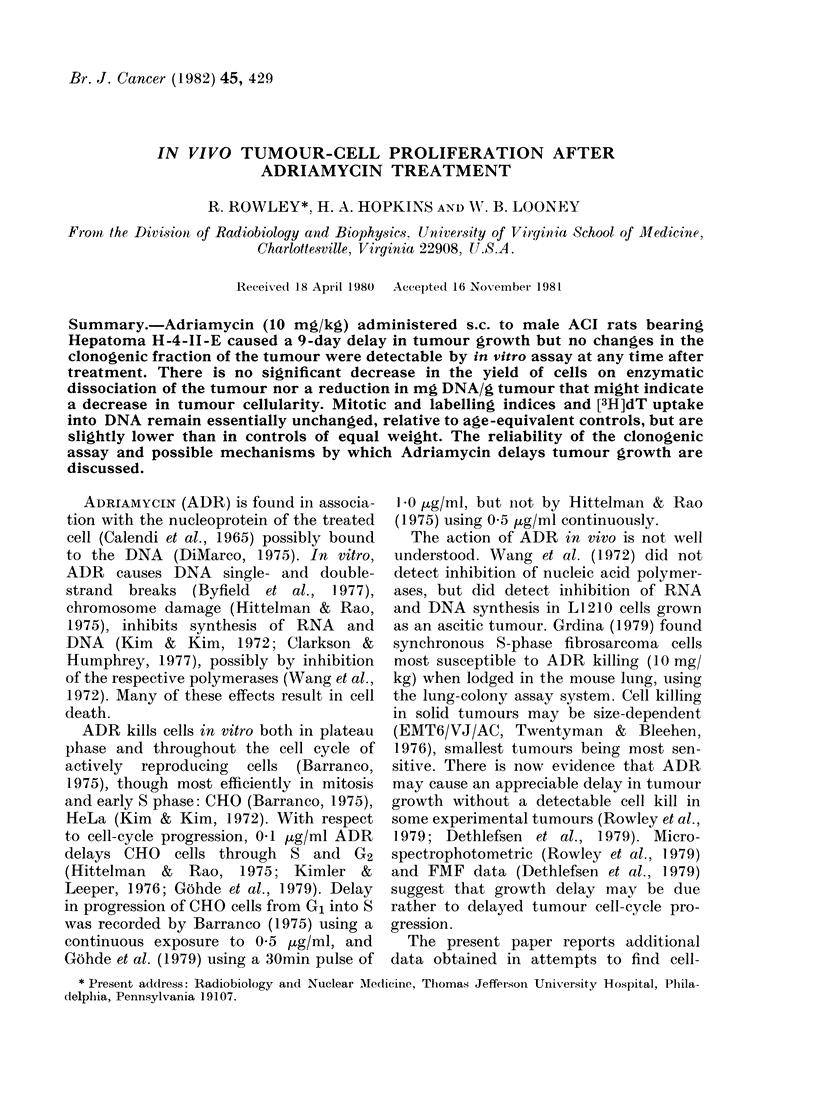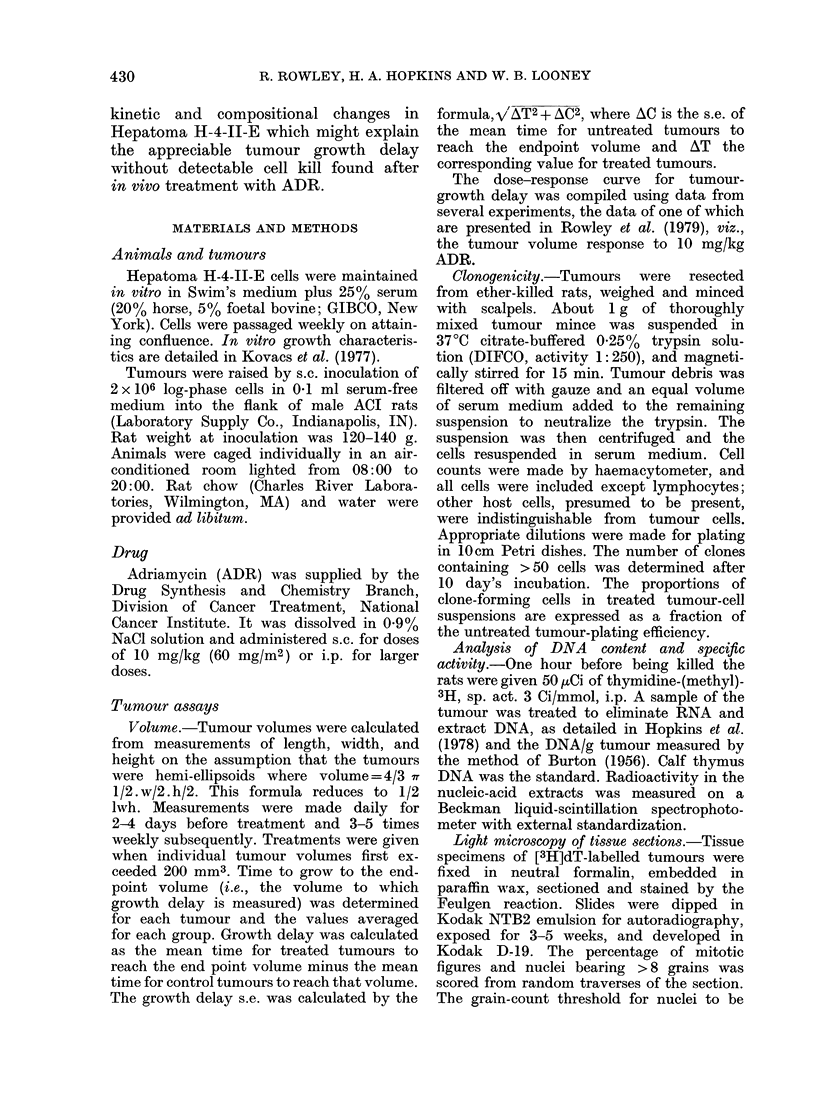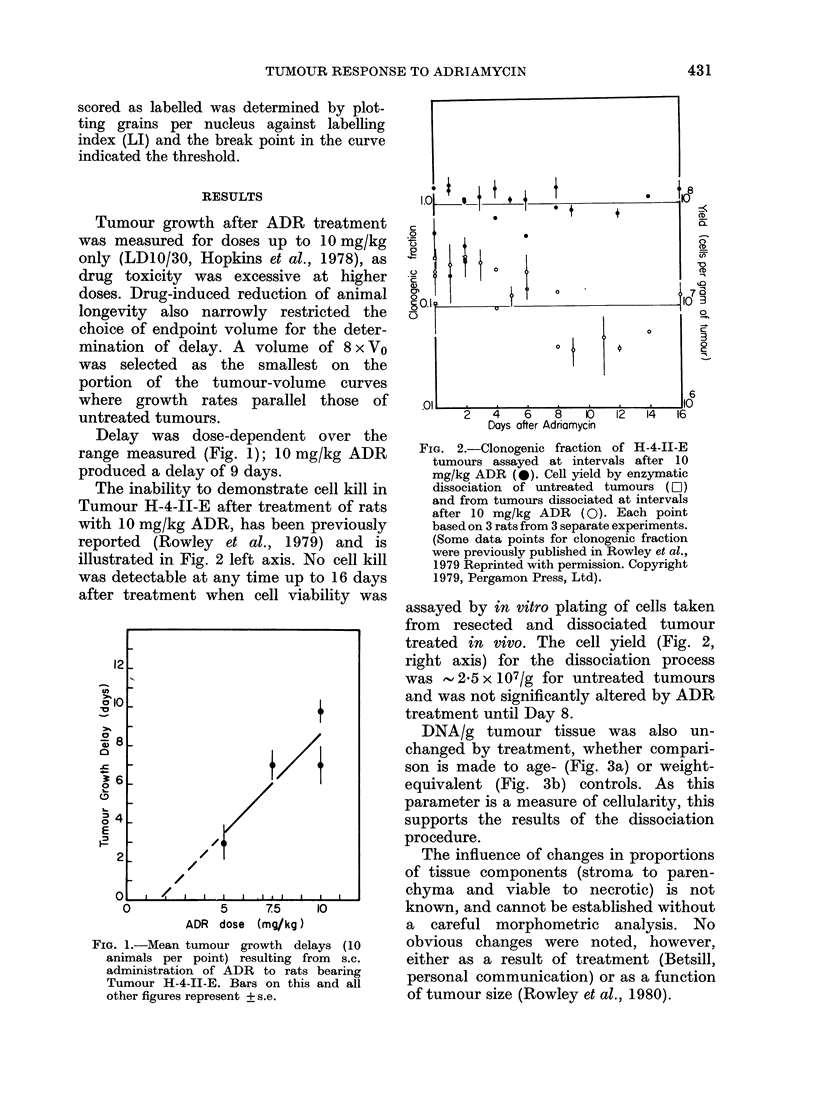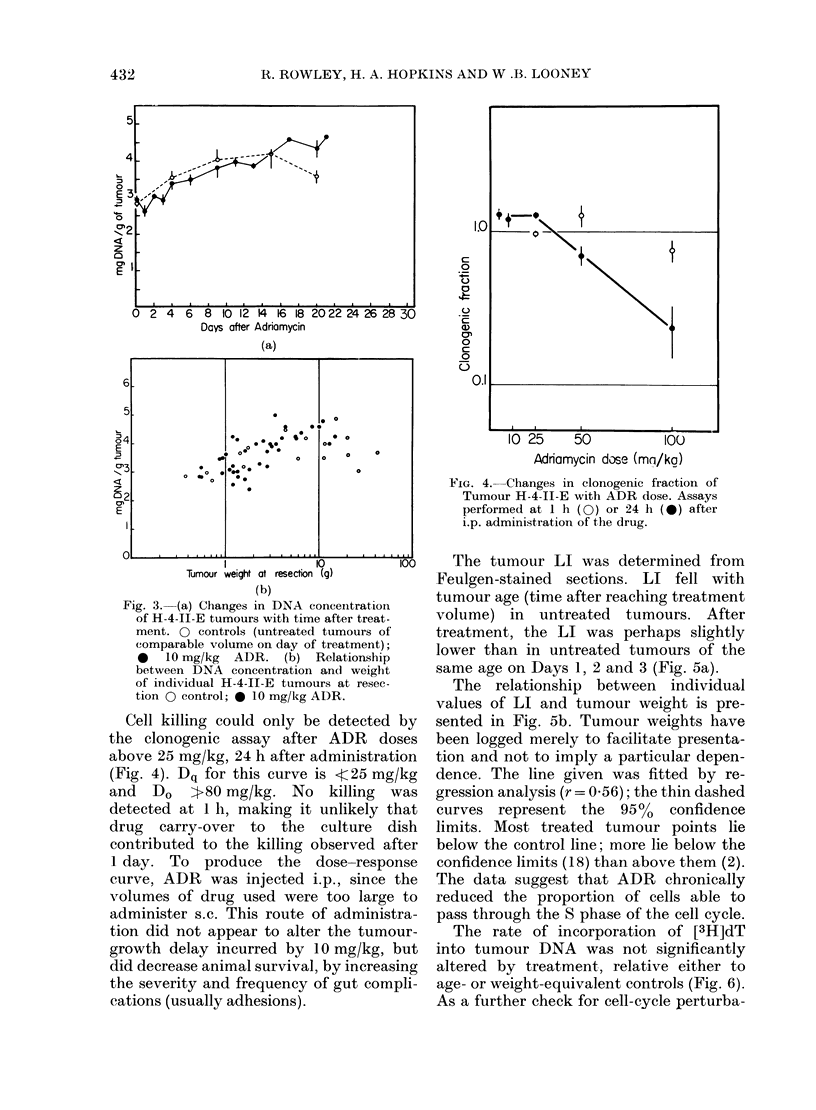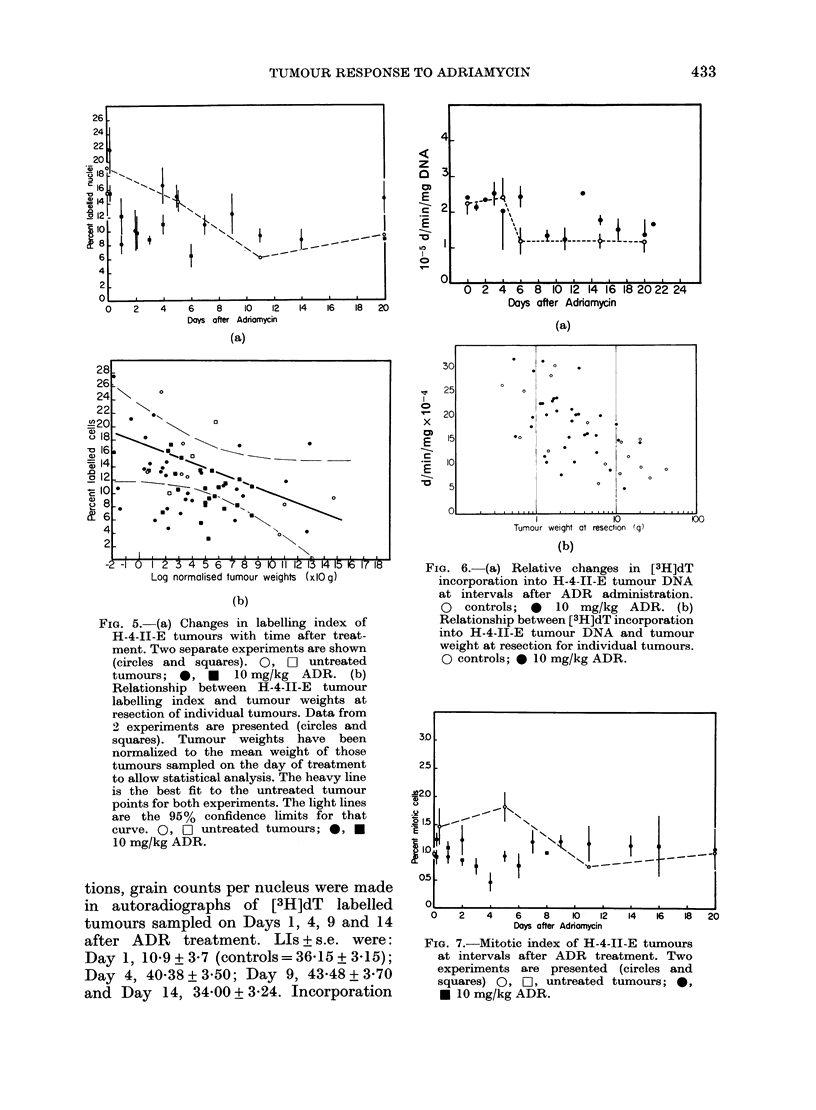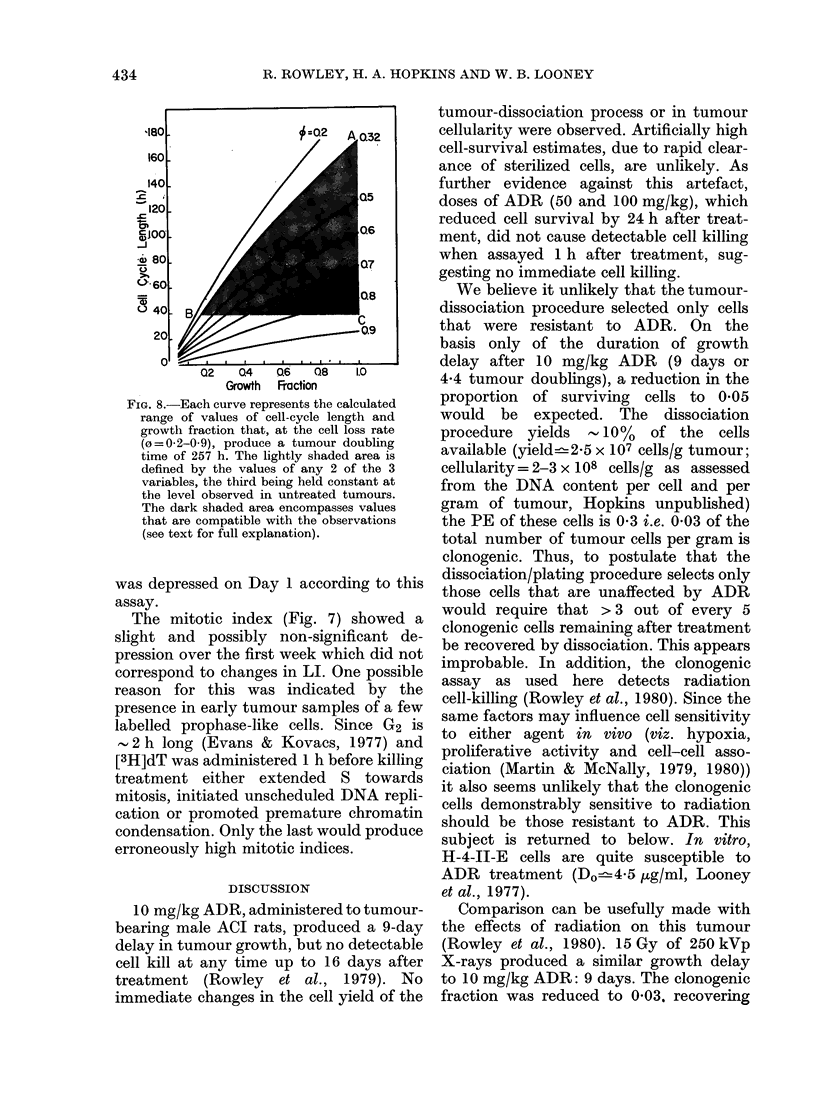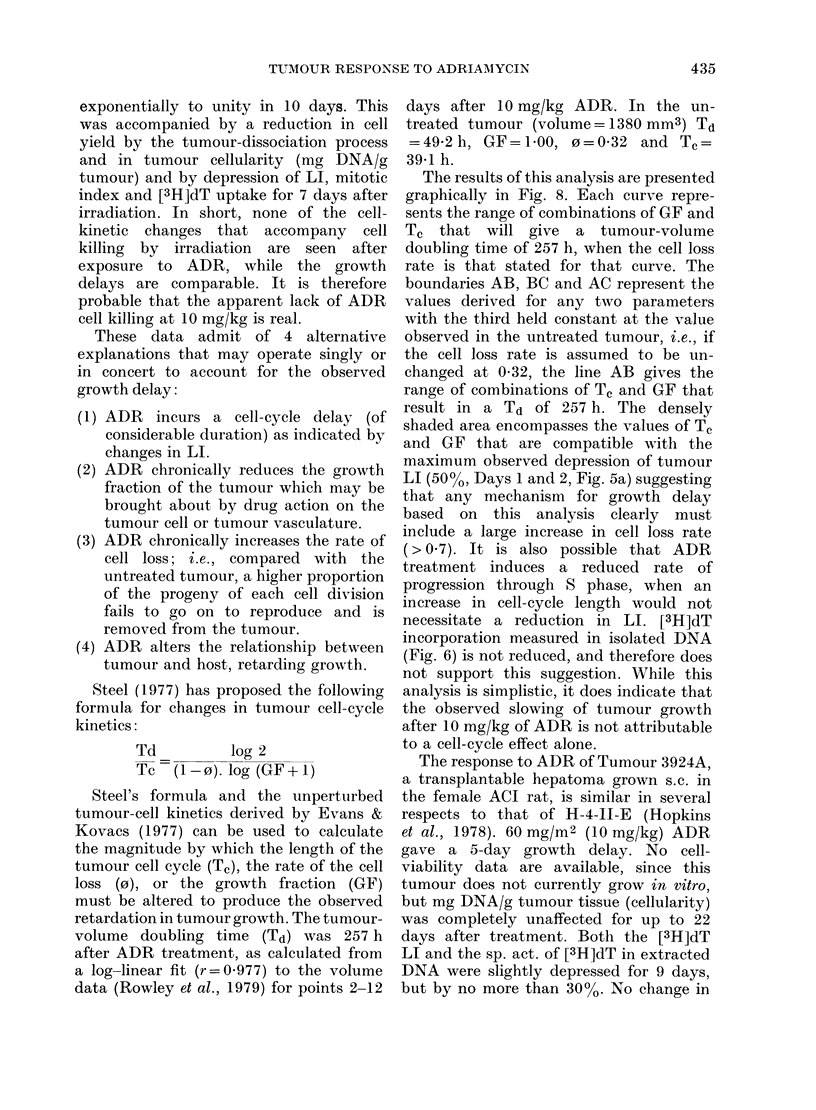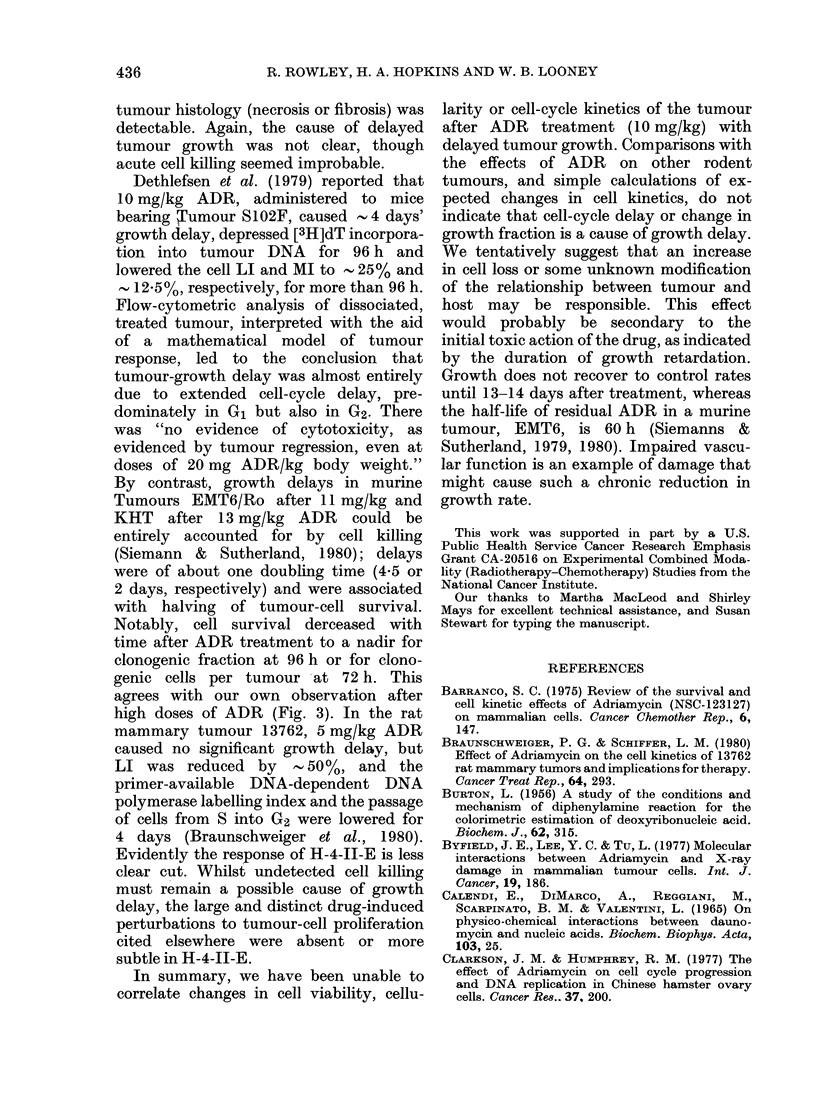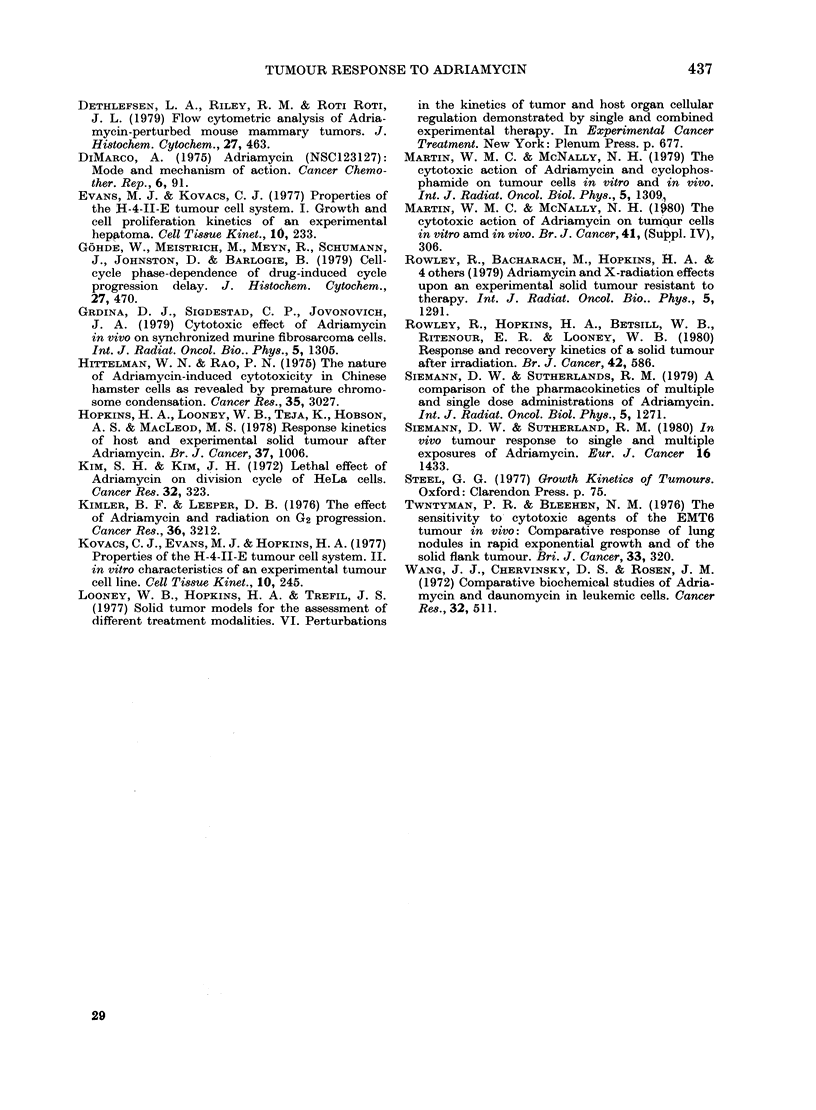# In vivo tumour-cell proliferation after adriamycin treatment.

**DOI:** 10.1038/bjc.1982.71

**Published:** 1982-03

**Authors:** R. Rowley, H. A. Hopkins, W. B. Looney

## Abstract

Adriamycin (10 mg/kg) administered s.c. to male ACI rats bearing Hepatoma H-4-II-E caused a 9-day delay in tumour growth but no changes in the clonogenic fraction of the tumour were detectable by in vitro assay at any time after treatment. There is no significant decrease in the yield of cells on enzymatic dissociation of the tumour nor a reduction in mg DNA/g tumour that might indicate a decrease in tumour cellularity. Mitotic and labelling indices and [3H]dT uptake into DNA remain essentially unchanged, relative to age-equivalent controls, but are slightly lower than in controls of equal weight. The reliability of the clonogenic assay and possible mechanisms by which Adriamycin delays tumour growth are discussed.


*Br. J. Cancer* (1982) **45**, 429

### *IN VIVO* TUMOUR-CELL PROLIFERATION AFTER ADRIAMYCIN TREATMENT

R. ROWLEY*, H. A. HOPKINS AND W. B. LOONEY

*From the Division of Radiobiology and Biophysics, University of Virginia School of Medicine, Charlottesville, Virginia* 22908, *U.S.A.*

Received 18 April 1980 Accepted 16 November 1981

**Summary.—Adriamycin (10 mg/kg) administered s.c. to male ACI rats bearing Hepatoma H-4-II-E caused a 9-day delay in tumour growth but no changes in the clonogenic fraction of the tumour were detectable by *in vitro* assay at any time after treatment. There is no significant decrease in the yield of cells on enzymatic dissociation of the tumour nor a reduction in mg DNA/g tumour that might indicate a decrease in tumour cellularity. Mitotic and labelling indices and [$^3$H]dT uptake into DNA remain essentially unchanged, relative to age-equivalent controls, but are slightly lower than in controls of equal weight. The reliability of the clonogenic assay and possible mechanisms by which Adriamycin delays tumour growth are discussed.**

ADRIAMYCIN (ADR) is found in association with the nucleoprotein of the treated cell (Calendi *et al.*, 1965) possibly bound to the DNA (DiMarco, 1975). *In vitro*, ADR causes DNA single- and double-strand breaks (Byfield *et al.*, 1977), chromosome damage (Hittelman & Rao, 1975), inhibits synthesis of RNA and DNA (Kim & Kim, 1972; Clarkson & Humphrey, 1977), possibly by inhibition of the respective polymerases (Wang *et al.*, 1972). Many of these effects result in cell death.

ADR kills cells *in vitro* both in plateau phase and throughout the cell cycle of actively reproducing cells (Barranco, 1975), though most efficiently in mitosis and early S phase: CHO (Barranco, 1975), HeLa (Kim & Kim, 1972). With respect to cell-cycle progression, 0·1 μg/ml ADR delays CHO cells through S and $G_2$ (Hittelman & Rao, 1975; Kimler & Leeper, 1976; Göhde *et al.*, 1979). Delay in progression of CHO cells from $G_1$ into S was recorded by Barranco (1975) using a continuous exposure to 0·5 μg/ml, and Göhde *et al.* (1979) using a 30min pulse of 1·0 μg/ml, but not by Hittelman & Rao (1975) using 0·5 μg/ml continuously.

The action of ADR *in vivo* is not well understood. Wang *et al.* (1972) did not detect inhibition of nucleic acid polymerases, but did detect inhibition of RNA and DNA synthesis in L1210 cells grown as an ascitic tumour. Grdina (1979) found synchronous S-phase fibrosarcoma cells most susceptible to ADR killing (10 mg/kg) when lodged in the mouse lung, using the lung-colony assay system. Cell killing in solid tumours may be size-dependent (EMT6/VJ/AC, Twentyman & Bleehen, 1976), smallest tumours being most sensitive. There is now evidence that ADR may cause an appreciable delay in tumour growth without a detectable cell kill in some experimental tumours (Rowley *et al.*, 1979; Dethlefsen *et al.*, 1979). Microspectrophotometric (Rowley *et al.*, 1979) and FMF data (Dethlefsen *et al.*, 1979) suggest that growth delay may be due rather to delayed tumour cell-cycle progression.

The present paper reports additional data obtained in attempts to find cell-

* Present address: Radiobiology and Nuclear Medicine, Thomas Jefferson University Hospital, Philadelphia, Pennsylvania 19107.

kinetic and compositional changes in Hepatoma H-4-II-E which might explain the appreciable tumour growth delay without detectable cell kill found after *in vivo* treatment with ADR.

### MATERIALS AND METHODS

### *Animals and tumours*

Hepatoma H-4-II-E cells were maintained *in vitro* in Swim's medium plus 25% serum (20% horse, 5% foetal bovine; GIBCO, New York). Cells were passaged weekly on attaining confluence. *In vitro* growth characteristics are detailed in Kovacs *et al.* (1977).

Tumours were raised by s.c. inoculation of $2 \times 10^6$ log-phase cells in 0·1 ml serum-free medium into the flank of male ACI rats (Laboratory Supply Co., Indianapolis, IN). Rat weight at inoculation was 120–140 g. Animals were caged individually in an air-conditioned room lighted from 08:00 to 20:00. Rat chow (Charles River Laboratories, Wilmington, MA) and water were provided *ad libitum.*

### *Drug*

Adriamycin (ADR) was supplied by the Drug Synthesis and Chemistry Branch, Division of Cancer Treatment, National Cancer Institute. It was dissolved in 0·9% NaCl solution and administered s.c. for doses of 10 mg/kg (60 mg/$m^2$) or i.p. for larger doses.

### *Tumour assays*

*Volume.*—Tumour volumes were calculated from measurements of length, width, and height on the assumption that the tumours were hemi-ellipsoids where volume $= 4/3\ \pi\ 1/2.w/2.h/2$. This formula reduces to 1/2 lwh. Measurements were made daily for 2–4 days before treatment and 3–5 times weekly subsequently. Treatments were given when individual tumour volumes first exceeded 200 $mm^3$. Time to grow to the endpoint volume (*i.e.*, the volume to which growth delay is measured) was determined for each tumour and the values averaged for each group. Growth delay was calculated as the mean time for treated tumours to reach the end point volume minus the mean time for control tumours to reach that volume. The growth delay s.e. was calculated by the formula, $\sqrt{\Delta T^2 + \Delta C^2}$, where $\Delta C$ is the s.e. of the mean time for untreated tumours to reach the endpoint volume and $\Delta T$ the corresponding value for treated tumours.

The dose–response curve for tumour-growth delay was compiled using data from several experiments, the data of one of which are presented in Rowley *et al.* (1979), *viz.*, the tumour volume response to 10 mg/kg ADR.

*Clonogenicity.*—Tumours were resected from ether-killed rats, weighed and minced with scalpels. About 1 g of thoroughly mixed tumour mince was suspended in 37°C citrate-buffered 0·25% trypsin solution (DIFCO, activity 1:250), and magnetically stirred for 15 min. Tumour debris was filtered off with gauze and an equal volume of serum medium added to the remaining suspension to neutralize the trypsin. The suspension was then centrifuged and the cells resuspended in serum medium. Cell counts were made by haemacytometer, and all cells were included except lymphocytes; other host cells, presumed to be present, were indistinguishable from tumour cells. Appropriate dilutions were made for plating in 10 cm Petri dishes. The number of clones containing $>50$ cells was determined after 10 day's incubation. The proportions of clone-forming cells in treated tumour-cell suspensions are expressed as a fraction of the untreated tumour-plating efficiency.

*Analysis of DNA content and specific activity.*—One hour before being killed the rats were given 50 $\mu$Ci of thymidine-(methyl)-$^3$H, sp. act. 3 Ci/mmol, i.p. A sample of the tumour was treated to eliminate RNA and extract DNA, as detailed in Hopkins *et al.* (1978) and the DNA/g tumour measured by the method of Burton (1956). Calf thymus DNA was the standard. Radioactivity in the nucleic-acid extracts was measured on a Beckman liquid-scintillation spectrophotometer with external standardization.

*Light microscopy of tissue sections.*—Tissue specimens of [$^3$H]dT-labelled tumours were fixed in neutral formalin, embedded in paraffin wax, sectioned and stained by the Feulgen reaction. Slides were dipped in Kodak NTB2 emulsion for autoradiography, exposed for 3–5 weeks, and developed in Kodak D-19. The percentage of mitotic figures and nuclei bearing $>8$ grains was scored from random traverses of the section. The grain-count threshold for nuclei to be scored as labelled was determined by plotting grains per nucleus against labelling index (LI) and the break point in the curve indicated the threshold.

### RESULTS

Tumour growth after ADR treatment was measured for doses up to 10 mg/kg only (LD10/30, Hopkins *et al.*, 1978), as drug toxicity was excessive at higher doses. Drug-induced reduction of animal longevity also narrowly restricted the choice of endpoint volume for the determination of delay. A volume of $8 \times V_0$ was selected as the smallest on the portion of the tumour-volume curves where growth rates parallel those of untreated tumours.

Delay was dose-dependent over the range measured (Fig. 1); 10 mg/kg ADR produced a delay of 9 days.

The inability to demonstrate cell kill in Tumour H-4-II-E after treatment of rats with 10 mg/kg ADR, has been previously reported (Rowley *et al.*, 1979) and is illustrated in Fig. 2 left axis. No cell kill was detectable at any time up to 16 days after treatment when cell viability was assayed by *in vitro* plating of cells taken from resected and dissociated tumour treated *in vivo*. The cell yield (Fig. 2, right axis) for the dissociation process was $\sim 2{\cdot}5 \times 10^7$/g for untreated tumours and was not significantly altered by ADR treatment until Day 8.

FIG. 1.—Mean tumour growth delays (10 animals per point) resulting from s.c. administration of ADR to rats bearing Tumour H-4-II-E. Bars on this and all other figures represent ±s.e.

FIG. 2.—Clonogenic fraction of H-4-II-E tumours assayed at intervals after 10 mg/kg ADR (●). Cell yield by enzymatic dissociation of untreated tumours (□) and from tumours dissociated at intervals after 10 mg/kg ADR (○). Each point based on 3 rats from 3 separate experiments. (Some data points for clonogenic fraction were previously published in Rowley *et al.*, 1979 Reprinted with permission. Copyright 1979, Pergamon Press, Ltd).

DNA/g tumour tissue was also unchanged by treatment, whether comparison is made to age- (Fig. 3a) or weight-equivalent (Fig. 3b) controls. As this parameter is a measure of cellularity, this supports the results of the dissociation procedure.

The influence of changes in proportions of tissue components (stroma to parenchyma and viable to necrotic) is not known, and cannot be established without a careful morphometric analysis. No obvious changes were noted, however, either as a result of treatment (Betsill, personal communication) or as a function of tumour size (Rowley *et al.*, 1980).

Fig. 3.—(a) Changes in DNA concentration of H-4-II-E tumours with time after treatment. ○ controls (untreated tumours of comparable volume on day of treatment); ● 10 mg/kg ADR. (b) Relationship between DNA concentration and weight of individual H-4-II-E tumours at resection ○ control; ● 10 mg/kg ADR.

FIG. 4.—Changes in clonogenic fraction of Tumour H-4-II-E with ADR dose. Assays performed at 1 h (○) or 24 h (●) after i.p. administration of the drug.

Cell killing could only be detected by the clonogenic assay after ADR doses above 25 mg/kg, 24 h after administration (Fig. 4). $D_q$ for this curve is $\nless$25 mg/kg and $D_0$ $\ngtr$80 mg/kg. No killing was detected at 1 h, making it unlikely that drug carry-over to the culture dish contributed to the killing observed after 1 day. To produce the dose–response curve, ADR was injected i.p., since the volumes of drug used were too large to administer s.c. This route of administration did not appear to alter the tumour-growth delay incurred by 10 mg/kg, but did decrease animal survival, by increasing the severity and frequency of gut complications (usually adhesions).

The tumour LI was determined from Feulgen-stained sections. LI fell with tumour age (time after reaching treatment volume) in untreated tumours. After treatment, the LI was perhaps slightly lower than in untreated tumours of the same age on Days 1, 2 and 3 (Fig. 5a).

The relationship between individual values of LI and tumour weight is presented in Fig. 5b. Tumour weights have been logged merely to facilitate presentation and not to imply a particular dependence. The line given was fitted by regression analysis ($r = 0{\cdot}56$); the thin dashed curves represent the 95% confidence limits. Most treated tumour points lie below the control line; more lie below the confidence limits (18) than above them (2). The data suggest that ADR chronically reduced the proportion of cells able to pass through the S phase of the cell cycle.

The rate of incorporation of [$^3$H]dT into tumour DNA was not significantly altered by treatment, relative either to age- or weight-equivalent controls (Fig. 6). As a further check for cell-cycle perturba-

(a)

(b)

FIG. 5.—(a) Changes in labelling index of H-4-II-E tumours with time after treatment. Two separate experiments are shown (circles and squares). ○, □ untreated tumours; ●, ■ 10 mg/kg ADR. (b) Relationship between H-4-II-E tumour labelling index and tumour weights at resection of individual tumours. Data from 2 experiments are presented (circles and squares). Tumour weights have been normalized to the mean weight of those tumours sampled on the day of treatment to allow statistical analysis. The heavy line is the best fit to the untreated tumour points for both experiments. The light lines are the 95% confidence limits for that curve. ○, □ untreated tumours; ●, ■ 10 mg/kg ADR.

tions, grain counts per nucleus were made in autoradiographs of [$^3$H]dT labelled tumours sampled on Days 1, 4, 9 and 14 after ADR treatment. LIs ± s.e. were: Day 1, 10·9 ± 3·7 (controls = 36·15 ± 3·15); Day 4, 40·38 ± 3·50; Day 9, 43·48 ± 3·70 and Day 14, 34·00 ± 3·24. Incorporation

(a)

(b)

FIG. 6.—(a) Relative changes in [$^3$H]dT incorporation into H-4-II-E tumour DNA at intervals after ADR administration. ○ controls; ● 10 mg/kg ADR. (b) Relationship between [$^3$H]dT incorporation into H-4-II-E tumour DNA and tumour weight at resection for individual tumours. ○ controls; ● 10 mg/kg ADR.

FIG. 7.—Mitotic index of H-4-II-E tumours at intervals after ADR treatment. Two experiments are presented (circles and squares) ○, □, untreated tumours; ●, ■ 10 mg/kg ADR.

FIG. 8.—Each curve represents the calculated range of values of cell-cycle length and growth fraction that, at the cell loss rate ($\phi$ = 0·2–0·9), produce a tumour doubling time of 257 h. The lightly shaded area is defined by the values of any 2 of the 3 variables, the third being held constant at the level observed in untreated tumours. The dark shaded area encompasses values that are compatible with the observations (see text for full explanation).

was depressed on Day 1 according to this assay.

The mitotic index (Fig. 7) showed a slight and possibly non-significant depression over the first week which did not correspond to changes in LI. One possible reason for this was indicated by the presence in early tumour samples of a few labelled prophase-like cells. Since $G_2$ is ~2 h long (Evans & Kovacs, 1977) and [$^3$H]dT was administered 1 h before killing treatment either extended S towards mitosis, initiated unscheduled DNA replication or promoted premature chromatin condensation. Only the last would produce erroneously high mitotic indices.

### DISCUSSION

10 mg/kg ADR, administered to tumour-bearing male ACI rats, produced a 9-day delay in tumour growth, but no detectable cell kill at any time up to 16 days after treatment (Rowley *et al.*, 1979). No immediate changes in the cell yield of the tumour-dissociation process or in tumour cellularity were observed. Artificially high cell-survival estimates, due to rapid clearance of sterilized cells, are unlikely. As further evidence against this artefact, doses of ADR (50 and 100 mg/kg), which reduced cell survival by 24 h after treatment, did not cause detectable cell killing when assayed 1 h after treatment, suggesting no immediate cell killing.

We believe it unlikely that the tumour-dissociation procedure selected only cells that were resistant to ADR. On the basis only of the duration of growth delay after 10 mg/kg ADR (9 days or 4·4 tumour doublings), a reduction in the proportion of surviving cells to 0·05 would be expected. The dissociation procedure yields ~10% of the cells available (yield≃2·5 × $10^7$ cells/g tumour; cellularity = 2–3 × $10^8$ cells/g as assessed from the DNA content per cell and per gram of tumour, Hopkins unpublished) the PE of these cells is 0·3 *i.e.* 0·03 of the total number of tumour cells per gram is clonogenic. Thus, to postulate that the dissociation/plating procedure selects only those cells that are unaffected by ADR would require that >3 out of every 5 clonogenic cells remaining after treatment be recovered by dissociation. This appears improbable. In addition, the clonogenic assay as used here detects radiation cell-killing (Rowley *et al.*, 1980). Since the same factors may influence cell sensitivity to either agent *in vivo* (*viz.* hypoxia, proliferative activity and cell–cell association (Martin & McNally, 1979, 1980)) it also seems unlikely that the clonogenic cells demonstrably sensitive to radiation should be those resistant to ADR. This subject is returned to below. *In vitro*, H-4-II-E cells are quite susceptible to ADR treatment ($D_0$≃4·5 μg/ml, Looney *et al.*, 1977).

Comparison can be usefully made with the effects of radiation on this tumour (Rowley *et al.*, 1980). 15 Gy of 250 kVp X-rays produced a similar growth delay to 10 mg/kg ADR: 9 days. The clonogenic fraction was reduced to 0·03, recovering exponentially to unity in 10 days. This was accompanied by a reduction in cell yield by the tumour-dissociation process and in tumour cellularity (mg DNA/g tumour) and by depression of LI, mitotic index and [$^3$H]dT uptake for 7 days after irradiation. In short, none of the cell-kinetic changes that accompany cell killing by irradiation are seen after exposure to ADR, while the growth delays are comparable. It is therefore probable that the apparent lack of ADR cell killing at 10 mg/kg is real.

These data admit of 4 alternative explanations that may operate singly or in concert to account for the observed growth delay:

(1) ADR incurs a cell-cycle delay (of considerable duration) as indicated by changes in LI.
(2) ADR chronically reduces the growth fraction of the tumour which may be brought about by drug action on the tumour cell or tumour vasculature.
(3) ADR chronically increases the rate of cell loss; *i.e.*, compared with the untreated tumour, a higher proportion of the progeny of each cell division fails to go on to reproduce and is removed from the tumour.
(4) ADR alters the relationship between tumour and host, retarding growth.

Steel (1977) has proposed the following formula for changes in tumour cell-cycle kinetics:

$$\frac{Td}{Tc} = \frac{\log 2}{(1-ø).\ \log\ (GF+1)}$$

Steel's formula and the unperturbed tumour-cell kinetics derived by Evans & Kovacs (1977) can be used to calculate the magnitude by which the length of the tumour cell cycle ($T_c$), the rate of the cell loss (ø), or the growth fraction (GF) must be altered to produce the observed retardation in tumour growth. The tumour-volume doubling time ($T_d$) was 257 h after ADR treatment, as calculated from a log–linear fit ($r = 0{\cdot}977$) to the volume data (Rowley *et al.*, 1979) for points 2–12 days after 10 mg/kg ADR. In the untreated tumour (volume = 1380 mm$^3$) $T_d$ = 49·2 h, GF = 1·00, ø = 0·32 and $T_c$ = 39·1 h.

The results of this analysis are presented graphically in Fig. 8. Each curve represents the range of combinations of GF and $T_c$ that will give a tumour-volume doubling time of 257 h, when the cell loss rate is that stated for that curve. The boundaries AB, BC and AC represent the values derived for any two parameters with the third held constant at the value observed in the untreated tumour, *i.e.*, if the cell loss rate is assumed to be unchanged at 0·32, the line AB gives the range of combinations of $T_c$ and GF that result in a $T_d$ of 257 h. The densely shaded area encompasses the values of $T_c$ and GF that are compatible with the maximum observed depression of tumour LI (50%, Days 1 and 2, Fig. 5a) suggesting that any mechanism for growth delay based on this analysis clearly must include a large increase in cell loss rate (> 0·7). It is also possible that ADR treatment induces a reduced rate of progression through S phase, when an increase in cell-cycle length would not necessitate a reduction in LI. [$^3$H]dT incorporation measured in isolated DNA (Fig. 6) is not reduced, and therefore does not support this suggestion. While this analysis is simplistic, it does indicate that the observed slowing of tumour growth after 10 mg/kg of ADR is not attributable to a cell-cycle effect alone.

The response to ADR of Tumour 3924A, a transplantable hepatoma grown s.c. in the female ACI rat, is similar in several respects to that of H-4-II-E (Hopkins *et al.*, 1978). 60 mg/m$^2$ (10 mg/kg) ADR gave a 5-day growth delay. No cell-viability data are available, since this tumour does not currently grow *in vitro*, but mg DNA/g tumour tissue (cellularity) was completely unaffected for up to 22 days after treatment. Both the [$^3$H]dT LI and the sp. act. of [$^3$H]dT in extracted DNA were slightly depressed for 9 days, but by no more than 30%. No change in tumour histology (necrosis or fibrosis) was detectable. Again, the cause of delayed tumour growth was not clear, though acute cell killing seemed improbable.

Dethlefsen *et al.* (1979) reported that 10 mg/kg ADR, administered to mice bearing Tumour S102F, caused ~4 days' growth delay, depressed [$^3$H]dT incorporation into tumour DNA for 96 h and lowered the cell LI and MI to ~25% and ~12·5%, respectively, for more than 96 h. Flow-cytometric analysis of dissociated, treated tumour, interpreted with the aid of a mathematical model of tumour response, led to the conclusion that tumour-growth delay was almost entirely due to extended cell-cycle delay, predominately in $G_1$ but also in $G_2$. There was "no evidence of cytotoxicity, as evidenced by tumour regression, even at doses of 20 mg ADR/kg body weight." By contrast, growth delays in murine Tumours EMT6/Ro after 11 mg/kg and KHT after 13 mg/kg ADR could be entirely accounted for by cell killing (Siemann & Sutherland, 1980); delays were of about one doubling time (4·5 or 2 days, respectively) and were associated with halving of tumour-cell survival. Notably, cell survival derceased with time after ADR treatment to a nadir for clonogenic fraction at 96 h or for clonogenic cells per tumour at 72 h. This agrees with our own observation after high doses of ADR (Fig. 3). In the rat mammary tumour 13762, 5 mg/kg ADR caused no significant growth delay, but LI was reduced by ~50%, and the primer-available DNA-dependent DNA polymerase labelling index and the passage of cells from S into $G_2$ were lowered for 4 days (Braunschweiger *et al.*, 1980). Evidently the response of H-4-II-E is less clear cut. Whilst undetected cell killing must remain a possible cause of growth delay, the large and distinct drug-induced perturbations to tumour-cell proliferation cited elsewhere were absent or more subtle in H-4-II-E.

In summary, we have been unable to correlate changes in cell viability, cellularity or cell-cycle kinetics of the tumour after ADR treatment (10 mg/kg) with delayed tumour growth. Comparisons with the effects of ADR on other rodent tumours, and simple calculations of expected changes in cell kinetics, do not indicate that cell-cycle delay or change in growth fraction is a cause of growth delay. We tentatively suggest that an increase in cell loss or some unknown modification of the relationship between tumour and host may be responsible. This effect would probably be secondary to the initial toxic action of the drug, as indicated by the duration of growth retardation. Growth does not recover to control rates until 13–14 days after treatment, whereas the half-life of residual ADR in a murine tumour, EMT6, is 60 h (Siemanns & Sutherland, 1979, 1980). Impaired vascular function is an example of damage that might cause such a chronic reduction in growth rate.

This work was supported in part by a U.S. Public Health Service Cancer Research Emphasis Grant CA-20516 on Experimental Combined Modality (Radiotherapy–Chemotherapy) Studies from the National Cancer Institute.

Our thanks to Martha MacLeod and Shirley Mays for excellent technical assistance, and Susan Stewart for typing the manuscript.

### REFERENCES

Barranco, S. C. (1975) Review of the survival and cell kinetic effects of Adriamycin (NSC-123127) on mammalian cells. *Cancer Chemother Rep.*, **6**, 147.

Braunschweiger, P. G. & Schiffer, L. M. (1980) Effect of Adriamycin on the cell kinetics of 13762 rat mammary tumors and implications for therapy. *Cancer Treat Rep.*, **64**, 293.

Burton, L. (1956) A study of the conditions and mechanism of diphenylamine reaction for the colorimetric estimation of deoxyribonucleic acid. *Biochem. J.*, **62**, 315.

Byfield, J. E., Lee, Y. C. & Tu, L. (1977) Molecular interactions between Adriamycin and X-ray damage in mammalian tumour cells. *Int. J. Cancer*, **19**, 186.

Calendi, E., DiMarco, A., Reggiani, M., Scarpinato, B. M. & Valentini, L. (1965) On physico-chemical interactions between daunomycin and nucleic acids. *Biochem. Biophys. Acta*, **103**, 25.

Clarkson, J. M. & Humphrey, R. M. (1977) The effect of Adriamycin on cell cycle progression and DNA replication in Chinese hamster ovary cells. *Cancer Res.*. **37**, 200.

DETHLEFSEN, L. A., RILEY, R. M. & ROTI ROTI, J. L. (1979) Flow cytometric analysis of Adriamycin-perturbed mouse mammary tumors. *J. Histochem. Cytochem.*, **27**, 463.

DIMARCO, A. (1975) Adriamycin (NSC123127): Mode and mechanism of action. *Cancer Chemother. Rep.*, **6**, 91.

EVANS, M. J. & KOVACS, C. J. (1977) Properties of the H-4-II-E tumour cell system. I. Growth and cell proliferation kinetics of an experimental hepatoma. *Cell Tissue Kinet.*, **10**, 233.

GÖHDE, W., MEISTRICH, M., MEYN, R., SCHUMANN, J., JOHNSTON, D. & BARLOGIE, B. (1979) Cell-cycle phase-dependence of drug-induced cycle progression delay. *J. Histochem. Cytochem.*, **27**, 470.

GRDINA, D. J., SIGDESTAD, C. P., JOVONOVICH, J. A. (1979) Cytotoxic effect of Adriamycin *in vivo* on synchronized murine fibrosarcoma cells. *Int. J. Radiat. Oncol. Bio.. Phys.*, **5**, 1305.

HITTELMAN, W. N. & RAO, P. N. (1975) The nature of Adriamycin-induced cytotoxicity in Chinese hamster cells as revealed by premature chromosome condensation. *Cancer Res.*, **35**, 3027.

HOPKINS, H. A., LOONEY, W. B., TEJA, K., HOBSON, A. S. & MACLEOD, M. S. (1978) Response kinetics of host and experimental solid tumour after Adriamycin. *Br. J. Cancer*, **37**, 1006.

KIM, S. H. & KIM, J. H. (1972) Lethal effect of Adriamycin on division cycle of HeLa cells. *Cancer Res.* **32**, 323.

KIMLER, B. F. & LEEPER, D. B. (1976) The effect of Adriamycin and radiation on $G_2$ progression. *Cancer Res.*, **36**, 3212.

KOVACS, C. J., EVANS, M. J. & HOPKINS, H. A. (1977) Properties of the H-4-II-E tumour cell system. II. *in vitro* characteristics of an experimental tumour cell line. *Cell Tissue Kinet.*, **10**, 245.

LOONEY, W. B., HOPKINS, H. A. & TREFIL, J. S. (1977) Solid tumor models for the assessment of different treatment modalities. VI. Perturbations in the kinetics of tumor and host organ cellular regulation demonstrated by single and combined experimental therapy. In *Experimental Cancer Treatment.* New York: Plenum Press. p. 677.

MARTIN, W. M. C. & MCNALLY, N. H. (1979) The cytotoxic action of Adriamycin and cyclophosphamide on tumour cells *in vitro* and *in vivo*. *Int. J. Radiat. Oncol. Biol. Phys.*, **5**, 1309,

MARTIN, W. M. C. & MCNALLY, N. H. (1980) The cytotoxic action of Adriamycin on tumour cells *in vitro* amd *in vivo*. *Br. J. Cancer*, **41**, (Suppl. IV), 306.

ROWLEY, R., BACHARACH, M., HOPKINS, H. A. & 4 others (1979) Adriamycin and X-radiation effects upon an experimental solid tumour resistant to therapy. *Int. J. Radiat. Oncol. Bio.. Phys.*, **5**, 1291.

ROWLEY, R., HOPKINS, H. A., BETSILL, W. B., RITENOUR, E. R. & LOONEY, W. B. (1980) Response and recovery kinetics of a solid tumour after irradiation. *Br. J. Cancer*, **42**, 586.

SIEMANN, D. W. & SUTHERLANDS, R. M. (1979) A comparison of the pharmacokinetics of multiple and single dose administrations of Adriamycin. *Int. J. Radiat. Oncol. Biol. Phys.*, **5**, 1271.

SIEMANN, D. W. & SUTHERLAND, R. M. (1980) *In vivo* tumour response to single and multiple exposures of Adriamycin. *Eur. J. Cancer* **16** 1433.

STEEL, G. G. (1977) *Growth Kinetics of Tumours.* Oxford: Clarendon Press. p. 75.

TWNTYMAN, P. R. & BLEEHEN, N. M. (1976) The sensitivity to cytotoxic agents of the EMT6 tumour *in vivo*: Comparative response of lung nodules in rapid exponential growth and of the solid flank tumour. *Bri. J. Cancer*, **33**, 320.

WANG, J. J., CHERVINSKY, D. S. & ROSEN, J. M. (1972) Comparative biochemical studies of Adriamycin and daunomycin in leukemic cells. *Cancer Res.*, **32**, 511.